# Preliminary Assessment of BNC Membranes as Solvent Delivery Systems for the Cleaning of Mural Paintings: Comparison with Traditional Gel Systems

**DOI:** 10.3390/gels12060551

**Published:** 2026-06-19

**Authors:** Francesco Menconi, Ulderico Santamaria, Alessandro Cardarelli, Eleonora Imperio, Sara Iafrate

**Affiliations:** 1 Mural Paintings Laboratory, Central Institute for Restoration (ICR)—Ministry of Culture, Via di San Michele 25, 00153 Rome, Italy; menconi.71183@icrestauro.com; 2 Department of Economics, Engineering, Society and Business Organization, Tuscia University, Largo dell’Università, 01100 Viterbo, Italy; santamaria@unitus.it (U.S.); a.cardarelli@unitus.it (A.C.); 3 Non-Destructive Testing Laboratory, Central Institute for Restoration (ICR)—Ministry of Culture, Via di San Michele 25, 00153 Rome, Italy; eleonoraimperio@yahoo.it

**Keywords:** bacterial nanocellulose, cleaning, cultural heritage, hydrogel, mural paintings, green materials, nanomaterials

## Abstract

Growing demand for greener and more sustainable materials in cultural heritage conservation has prompted the investigation of bio-based alternatives for cleaning applications. This study presents a preliminary evaluation of bacterial nanocellulose (BNC) membranes for the removal of acrylic resins from mural paintings, comparing commercial medical-grade and laboratory-produced BNC with conventional gel systems under simulated application conditions. Both BNC types were characterized in terms of composition, pH, electrical conductivity, Water Holding Capacity and Water Retention Rate. Acetone loading via solvent exchange was assessed by thermogravimetric analysis (TGA), while mechanical behavior before and after solvent loading was evaluated through tensile testing and optical density measurements of the immersion media. The performance of BNCs and reference delivery systems was comparatively assessed in terms of solvent retention, solvent penetration depth into the substrate and residue release. Cleaning performance was investigated through FTIR spectroscopy and semi-quantitative image analysis as indirect indicators of residual resin content, on both mock-up samples and in situ applications. Under the tested conditions, both BNC membranes were compatible with acetone loading and maintained mechanical integrity after solvent exposure. FTIR analysis showed a reduction in the acrylic carbonyl band after treatment with acetone-loaded BNC, which exhibited greater solvent diffusion depth; the underlying removal mechanism, including the possible contribution of solvent-driven redistribution phenomena, remains to be clarified. Differences in reproducibility were observed between medical-grade and laboratory-produced BNC. Overall, the study provides experimental data contributing to the assessment of BNC membranes as bio-based solvent delivery systems for conservation practice.

## 1. Introduction

The cleaning treatment of painted artworks represents a crucial issue in conservative intervention. It consists of removing layers of superimposed substances from the painted surface to regain aesthetic readability or to eliminate potentially harmful substances.

When applying cleaning agents on a painted surface, the use of thickeners and appropriate solvent delivery system, in particular gel systems, can guarantee a more reliable and safer action compared to neat solvents [1]. Their ability to retain solvents and reduce evaporation prolongs solvent–surface contact time, promoting the solubilization of undesired substances and thereby enhancing treatment efficacy. Furthermore, by limiting uncontrolled diffusion into the substrate, they can improve treatment selectivity and enable the gradual removal of unwanted materials through consecutive applications. However, their use on highly porous surfaces, such as mural paintings, raises concern about the possible release of residues from the delivery medium on the painted surface. These could be responsible for the long-term alteration of the original materials.

A wide range of gels can be used as delivery media for cleaning applications. These materials can be broadly classified into two main categories—chemical and physical gels [2]—according to the type of polymeric interactions and gelation mechanism involved [2,3,4,5]. Chemical gels are formed through covalent crosslinking, resulting in permanent, stable networks [6,7,8], whereas physical gels are based on reversible, non-covalent interactions [9]. Within these classes, both natural and synthetic materials are included [2,10], as well as gels exhibiting highly diverse physicochemical properties in terms of viscosity, retention capacity, and elasticity [11,12,13].

The growing demand for environmentally friendly products has stimulated the search for green materials in the field of conservation, favoring those derived from renewable sources and more sustainable methods. Within this framework, several nanocellulose-based materials—such as cellulose nanofibrils (CNFs), cellulose nanocrystals (CNCs) [14,15], and bacterial nanocellulose (BNC)—have been introduced into restoration practice for a wide range of applications [14,16,17,18,19,20].

BNC is a natural polymer biosynthesized via aerobic fermentation by various bacterial strains, especially acetic acid bacteria [21,22,23]. It is characterized by high crystallinity (84–89%), high polymerization degree (up to 20,000), a large aspect ratio and greater purity compared to plant-derived cellulose, due to the absence of lignin, hemicellulose, and other impurities [24,25,26]. It is produced intracellularly and secreted as fibrils composed by linear chains of cellobiose, which assemble in a three-dimensional, felt-like network stabilized by hydrogen bonds [24,25,26].

This unique structure imparts remarkable properties to the polymer matrix, potentially making it a highly promising material for cleaning applications. These include exceptional Water Holding Capacity and distinctive physical–mechanical characteristics [24,25,26].

**Figure 1:**
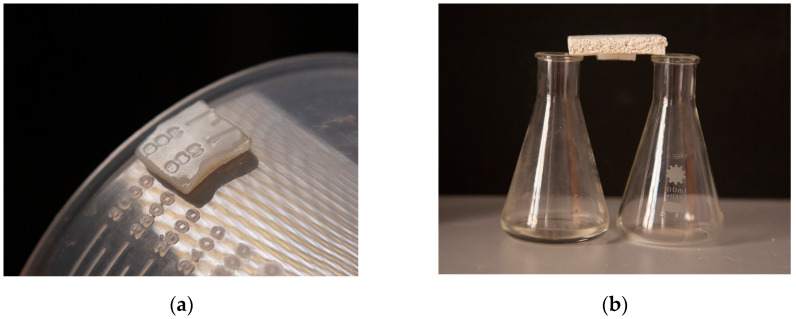
(**a**) BNC membrane observed in raking light after application on an irregular surface: the membrane accurately conforms to and replicates the substrate’s surface morphology; (**b**) BNC membrane applied to a mortar specimen positioned face down: the membrane remains firmly adhered to the plaster surface.

Moreover, BNC membranes show the ability to adapt to irregular surfaces (Figure 1a) and to remain stably adhered to the substrate during application on vertical and overhead surfaces (Figure 1b), without exhibiting tackiness. Notably, this adhesion behavior and the cohesive forces within the fiber network may facilitate the removal of the gel and minimize the risk of residual material on the painted surface, thereby reducing the need for additional clearance steps [27,28,29,30].

Despite these characteristics, its use as a delivery system for cleaning application has been explored in only a very limited number of studies [31,32,33,34,35].

Scientific research has optimized methods to scale up nanocellulose production and identified highly productive bacterial strains, such as *Komagataeibacter xylinus* [36,37,38,39], as well as effective culture media (e.g., *Hestrin–Schramm* medium) [40] and growing conditions [37,41,42,43], which ensure high cellulose yields. However, the reliance on specific and costly raw materials still makes this production process expensive, environmentally burdensome [37], and difficult to apply on a large scale, such as in the conservation of mural paintings. To address these limitations, recent studies have explored more sustainable and cost-effective production strategies [37,44,45,46,47]. Particular attention has been given to the reuse of BNC derived from food industry waste streams [48,49,50,51,52,53]. Among these approaches, the use of kombucha cultures, or SCOBY (Symbiotic Culture of Bacteria and Yeast), obtained through the fermentation of sweetened tea, has emerged as a promising low-cost and environmentally friendly alternative for producing BNC membranes [54,55]. This method offers advantages in terms of accessibility, scalability, and reproducibility, making it particularly attractive for conservation-oriented applications.

Building on these premises, this study compares the performance of a medical-grade, commercially available BNC membrane (NS) with that of a laboratory-produced BNC, obtained through the sterilization and purification of kombucha-derived SCOBY (NK), for the cleaning of mural paintings.

More specifically, the aim of the study was to develop an effective cleaning protocol for the treatment of the 12th-century frescoes in the crypt of the Sanctuary of Santa Maria del Piano in Ausonia (Frosinone, Italy), selected as a case study. These paintings are affected by the presence of a substantial acrylic resin layer—Paraloid B72 (EM-MA copolymer)—applied in the 1970s. The cleaning treatment consists of removing the acrylic film from the painted surface while minimizing mechanical stress on the paint layer and preventing the diffusion of the solubilized resin into the underlying plaster during the cleaning process. To enhance the solubilization of the acrylic film, an appropriate solvent, i.e., acetone, was combined with different delivery systems, including both conventional gels and BNC membranes, to identify the one providing the best performance. The cleaning procedure was limited to the simple application of a cleaning system on the painted surface, without subsequent refinement with swabs to avoid mechanical stress of the paint layer.

The first phase of research focused on the characterization of the two different BNC hydrogels, determining their composition, purity and compatibility with fresco substrate through pH, conductivity and ATR-FTIR measurement. Physicochemical properties were also investigated by gravimetric analyses to determine the Water Holding Capacity (WHC) and Water Release Rate (WRR). In addition, the loading capacity of BNC membranes with respect to the selected solvent for cleaning treatment (acetone) was assessed. A dedicated solvent-loading protocol, based on a solvent-exchange method [56], was used. Building on previous studies in which solvent exchange in hydrogels has been investigated by thermogravimetric techniques [57,58,59,60], this work employs thermogravimetric and differential scanning calorimetry (TG–DSC) to evaluate the effectiveness of solvent loading into BNC membranes from a conservation-oriented perspective. Specifically, thermal analysis was used to monitor the effectiveness of a water-to-ethanol-to-acetone exchange sequence and to compare the resulting solvent loading with the operational requirements of conservation treatments. The integrity of the membranes after solvent loading was indirectly evaluated through optical density measurements of solvents used in the procedure. Changes in the mechanical properties of the membranes resulting from acetone loading were assessed through tensile tests.

The second phase of the study was devoted to the evaluation of the operational properties of both BNCs comparatively assessed against a selection of gel-based systems currently adopted in conservation practice. Tests were conducted on mock-up samples reproducing the case study.

The reference delivery systems were selected to represent a broad range of materials used in conservation, including both natural and synthetic matrices with different gelation mechanisms, structures, and solvent interactions. Systems with varying retention properties were included, from highly retentive rigid gels to more diffusive materials (Table 1).

Two agar-based systems were selected: a thermoreversible rigid polysaccharide gel derived from agar–agar, Agar Art^®^ (AG); and a homogenized rigid gel obtained by double cooking and grounding of AgarArt, NEVEK^®^ (NV). In contrast, a poly(vinyl alcohol)-based system was chosen as a synthetic benchmark: a highly viscous polymeric dispersion (HVPD) based on a physically linked poly(vinyl alcohol)–borate network (PB). Finally, paper pulp (ARBOCEL^®^ BWW-40) was used as a low-retentive reference material (PC), characterized by limited solvent confinement and rapid release, serving as a baseline for evaluating solvent diffusion and evaporation without a structured gel network.

Particular attention was devoted to the investigation of the confinement behavior of each system with respect to acetone, evaluating the ability to retard its evaporation and limit its diffusion into the substrate.

Surface changes induced by the treatment were assessed by UV examination of mock-up samples after application, and fluorescent areas were subsequently characterized by DRIFT-FTIR analysis to evaluate their possible attribution to gel residues. Cleaning performance was evaluated through a comparative assessment of resin removal from plaster substrates using fluorescence image analysis of the mock-up specimens before and after treatment, using Rhodamine as a fluorescent tracer embedded in the acrylic polymer matrix. The results were further supported by portable FT-IR spectroscopy performed on the specimens both before and after the cleaning treatments, as an indirect and complementary method for monitoring changes in surface resin content.

In addition, FT-IR analyses were also conducted in situ to verify the applicability and effectiveness of the proposed cleaning systems under real conservation conditions.

The experimental setup is summarized in Table 2.

This study expands current knowledge on the application of bacterial nanocellulose in cultural heritage conservation by investigating its use for the removal of acrylic films from painted mortar substrates. It provides a comparative evaluation of two types of BNC—a medical-grade material and a laboratory-produced variant—alongside other commonly used delivery systems under realistic application conditions. This approach offers conservators a more informed basis for selecting appropriate cleaning materials and supports the exploration of greener alternatives for the conservation of architectural surfaces. Beyond BNC performance assessment, the study also examines aspects that have received limited attention in previous research within the field of cultural heritage conservation, including solvent diffusion within the substrate, compatibility in terms of pH and conductivity, and changes in mechanical behavior after acetone loading. At the same time, it identifies open questions concerning potential residue formation and the mechanisms governing gel-assisted cleaning processes.

## 2. Results and Discussion

### 2.1. Characterization of BNC Membranes

#### 2.1.1. Chemical Compatibility Assessment for Conservation Applications

Both BNC membranes were analyzed to assess their chemical composition and their suitability for conservation applications. These tests were also aimed at validating the purification process of the laboratory-produced BNC in comparison with the medical-grade BNC, used as a reference. The absence of residues of alkaline substances is essential to prevent the release of potentially undesirable compounds onto the painted surface during solvent delivery.

**Figure 2:**
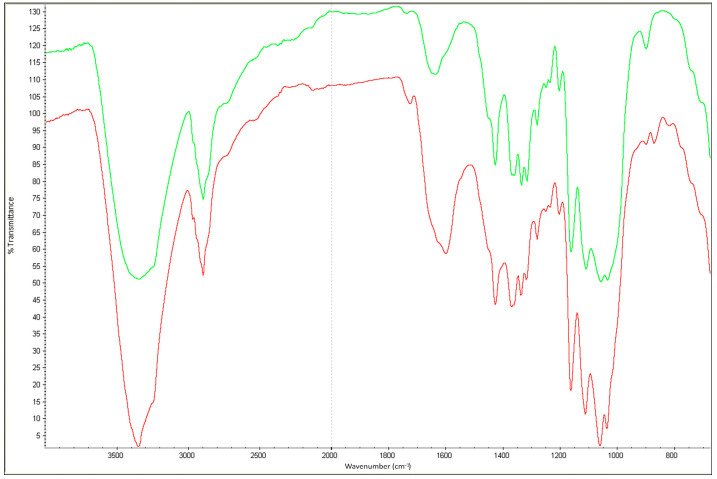
FTIR spectra of NS (green line) and NK membranes (red line).

FTIR measurements conducted on the two previously dehydrated BNC membranes (Figure 2) show substantially overlapping spectra, with characteristic peaks typical of cellulose reported in the literature (3451 cm^−1^ and 2899 cm^−1^ peaks for O-H and C-H stretching vibrations; 1644 cm^−1^ O-H vibration of absorbed water; 1382 cm^−1^ peak for C-H and C-O vibrations; 1060 cm^−1^ for C-O-C in pyranose ring) [61]. The similarity of the spectra suggests that the laboratory production and purification process yields a cellulose material comparable to the medical-grade reference and reveals no evidence of major chemical impurities or structural alterations detectable by FTIR, although the presence of low-molecular-weight residual compounds cannot be excluded [62].

To assess the possible presence of soluble trace ionic species originating from the purification process, pH and conductivity measurements were additionally performed on the soaking water after immersion of the BNC membranes in demineralized water for 24 h under continuous stirring. This extraction procedure was designed to promote the release of any residual water-soluble compounds that could be relevant for conservation applications.

Consistently, pH and conductivity measurements of the soaking water showed near-neutral pH and conductivity values, comparable to those of the demineralized water used for the extraction, suggesting that the release of water-soluble residues from the membranes was limited under the tested conditions, and supporting the effectiveness of the purification procedure (Table 3).

#### 2.1.2. Water Holding Capacity (WHC) and Water Release Rate (WRR)

The Water Holding Capacity (WHC) and Water Release Rate (WRR) of both BNC membranes were measured to compare their properties in terms of solvent uptake and retention. These properties were then correlated with the swelling behavior of the two membranes.

Both membranes exhibit comparable behavior in terms of WHC and WRR, with values consistent with those reported in previous studies [33,35,63]. A greater data dispersion of laboratory-produced BNC (NK) is observed, likely attributable to the lower degree of control over the growth conditions (Table 4).

**Figure 3:**
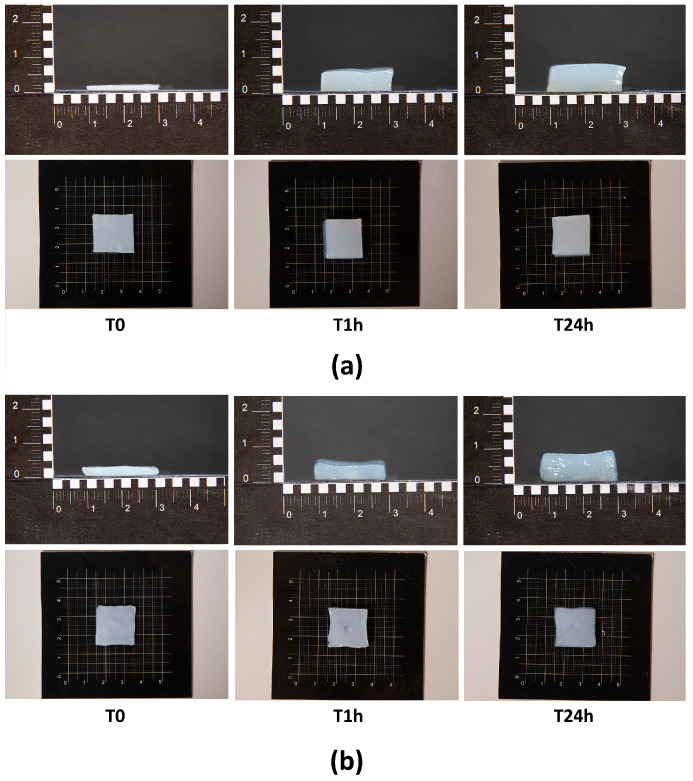
NS (**a**) and NK (**b**) dimensional changes during swelling at 0 h, 1 h, and 24 h: the first and third rows show vertical swelling, while the second and fourth rows illustrate in-plane dimensional changes.

BNC membranes also exhibit similar swelling behavior during hydration (Table 5), with significant vertical swelling and minimal in-plane dimensional changes (Figure 3 and Figure S1). This is due to their layered structure, where fibrils randomly displace parallel to the surface, with denser regions alternating looser ones that promote liquid retention and favor expansion mainly in the vertical direction [64]. NS shows a more pronounced and rapid swelling after 1 h immersion, compared to NK. After 24 h immersion the swelling of the two membranes is comparable.

This characteristic is especially beneficial in reducing mechanical stress and minimizing the risk of damage to the pictorial surface caused by shrinkage during drying of the membrane potentially occurring during the cleaning procedure.

#### 2.1.3. Solvent-Loading Assessment

Due to the difficulty of hydrophilic BNC membranes in directly uptaking solvents less polar than water, such as acetone, a solvent-loading process is not straightforward. For this reason, a solvent-exchange procedure starting from water-loaded BNC membranes was applied. The samples were prepared through a stepwise process, involving sequential immersion in solvents or solvent mixtures with progressively decreasing polarity, following the sequence water–water/ethanol–ethanol–ethanol/acetone–acetone. TG and DTG profiles of BNC membrane samples loaded with water (_H_2_O), ethanol (_AL), and acetone (_AC) were analyzed to evaluate the effectiveness of the exchange procedure and to monitor the solvent-release behavior at each step. The interpretation of events observed in the DTG curves was supported by DSC curves (Figure S2).

**Figure 4:**
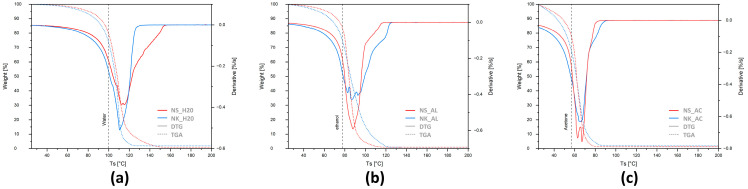
TG and DTG curves of NK and NS loaded with water (**a**), ethanol (**b**) and acetone (**c**) following the solvent-exchange process.

The main thermal parameters obtained from triplicate measurements are listed in Table S1. The results reveal some differences in the solvent release behavior of the BNC membranes, enabling comparison of evaporation onset, peak temperature, endset temperature, and overall peak profile (Figure 4). These features were used as comparative indicators of the interaction between the liquid phase and the BNC network, as well as of the progression of the solvent-exchange process. However, DTG peak shape and peak broadening were not considered direct evidence of discrete solvent populations, as they may also reflect differences in release kinetics, membrane structure, and matrix–solvent interactions.

Overall, all BNC samples exhibit delayed solvent evaporation relative to the boiling temperatures of the corresponding free solvent, with average increases of 12 °C for water, 9.5 °C for ethanol and 13 °C for acetone. This behavior suggests that the BNC network contributes to solvent retention by slowing down evaporation compared with the free liquid phase.

The two water-loaded BNC membranes (Figure 4a) showed slightly different thermal behaviors. NS_H_2_O exhibits a more pronounced delay in evaporation, with a broad asymmetric DTG peak centered at 114 °C, an onset at 60 °C, and mass loss continuing up to 155 °C. By contrast, NK_H_2_O shows an earlier and narrower peak centered at approximately 110 °C. These differences indicate that the two membranes interact differently with water during heating, likely reflecting variations in their nanofibrillar network organization and retention behavior.

The NS_AL sample exhibits a sharp, nearly symmetrical DTG peak at 87.6 °C, consistent with ethanol being the main volatile component after the water-to-ethanol exchange step. This profile suggests an efficient replacement of water by ethanol in the NS membrane. Conversely, NK_AL exhibits a broader mass loss profile in the same temperature range, with less regular features and a contribution extending toward higher temperatures. This behavior indicates a less uniform release process and suggests that the exchange step was less complete in NK than in NS. The signal observed around 100 °C is compatible with the presence of a residual water contribution, although this attribution should be considered indicative rather than definitive in the absence of complementary quantitative analysis (Figure 4b).

In the acetone-loaded samples (Figure 4c), both NS_AC and NK_AC exhibit a well-defined main DTG peak in the 62–66 °C range with an early mass-loss onset at approximately 56–58 °C, close to the boiling point of free acetone (56 °C). This behavior suggests that acetone was effectively introduced into both BNC membranes and was readily released during heating. NK_AC shows a post-peak shoulder in the 75–78 °C range, suggesting a minor contribution from residual ethanol retained from the previous exchange step, whereas this contribution was less evident in NS_AC. No water-related events were detected within the sensitivity of TG–DSC, suggesting that the exchange protocol effectively reduced the water fraction under the tested conditions. For the purposes of the present application, this reduction was considered adequate to obtain acetone-loaded BNC membranes suitable for use as solvent-delivery systems in cleaning tests, where the main requirement was to minimize the presence of water sufficiently to preserve the solvent action of acetone during application.

The ability of nanocellulose to incorporate solvents less polar than water is likely related to its amphiphilic nature. Nanofibrils can orient within the three-dimensional network, exposing different regions of the cellulose chains that can alternatively promote interaction with solvents of different polarity, thereby contributing to solvent uptake and retention [65]. Overall, TG–DSC analysis confirmed that the stepwise water-to-ethanol-to-acetone exchange protocol provides an effective route for preparing acetone-loaded BNC membranes for conservation-oriented cleaning applications, while allowing comparison between the commercial medical-grade membrane and the laboratory-produced membrane.

#### 2.1.4. Tensile Properties

Tensile tests were performed on both NK and NS membranes loaded with water and acetone to characterize their mechanical behavior and to evaluate the effect of solvent loading on their properties.

The results (Table 6) indicate that acetone-loaded samples exhibit higher tensile strength (*σ*) and maximum force (*Fmax*), together with lower elongation at break (*ε*), compared with water-loaded samples. In particular, NK acetone-treated membranes show the highest average tensile strength (*σ* = 2.97 and 1.63 MPa for the reported conditions).

Water-loaded samples display lower tensile strength and higher strain at break (*ε* ~ 20–22%), indicating a more compliant mechanical response. This behavior is consistent with the well-known plasticizing effect of water in nanocellulose-based hydrogels, as previously reported in the literature [66,67], where water promotes fibril mobility by modulating intermolecular interactions within the network.

In contrast, acetone-loaded samples show a stiffer response. This trend may be associated with changes induced by solvent exchange within the nanofibrillar network, as also reported for hydrogel systems subjected to water–solvent replacement, where modifications in the surrounding liquid environment can influence polymer–polymer interactions and the overall network response [68,69,70].

Although the mechanical data suggest a solvent-dependent variation in the behavior of the nanocellulosic network, their interpretation at the molecular level remains open. From an application-oriented perspective, these results indicate that the mechanical response of the BNC systems is not an intrinsic, fixed property, but is influenced by the solvent loading conditions, which may modulate the balance between stiffness and deformability of the gel. This aspect should therefore be carefully considered when selecting solvent–gel combinations for practical conservation treatments, as it may affect handling and interfacial contact with the substrate.

#### 2.1.5. Mechanical Integrity in Solvents

The mechanical integrity of both BNC membranes in different solvents was indirectly assessed through optical density analysis of the media used during the solvent-loading procedure (water, ethanol, and acetone), following prolonged immersion and subsequent agitation. No visible deposits were observed in the immersion media prior to measurement.

**Figure 5:**
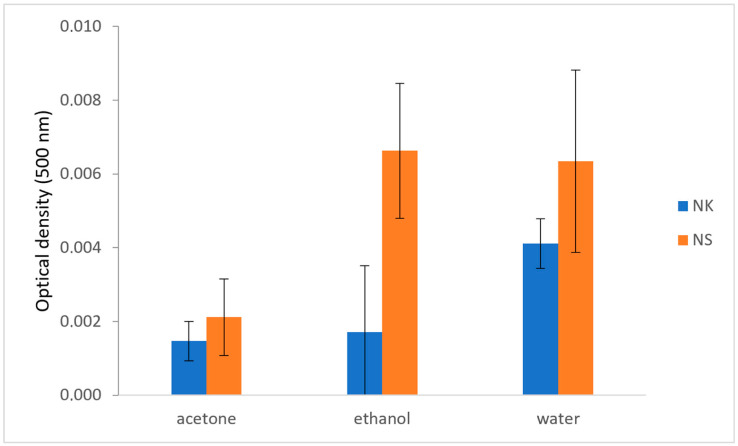
Optical density of water, ethanol, and acetone after immersion of NK and NS samples, reported as mean absorbance values at 500 nm.

Results are reported as mean optical density at 500 nm for each batch of samples (Figure 5).

Overall, the results show absorbance values close to zero for all solvents and for both BNC types, indicating negligible levels of suspended material in the immersion media. These findings suggest that the membranes maintain mechanical integrity after solvent loading and prolonged immersion in acetone and ethanol.

NK samples consistently exhibited slightly lower absorbance values than NS samples under all tested conditions. Minor differences were observed among the solvents, with marginally higher absorbance values recorded for water and ethanol than for acetone. The lower values observed in acetone may be tentatively associated with changes in the interactions within the cellulose network induced by solvent exchange. The removal of water reduced plasticization effects and promoted closer polymer–polymer interactions within the network [70]. While this interpretation remains speculative, it is consistent with mechanical test results reported in Section 2.1.4.

### 2.2. Comparative Tests

#### 2.2.1. Solvent Retention Test

The capacity of BNC membranes and reference products to retain acetone (AC), thereby delaying its evaporation, was evaluated to compare their performance. For gel systems requiring water for network formation, the maximum amount of acetone that does not affect the gelation mechanism was added, as reported in the respective technical data sheets and technical literature. The aim of this test was to assess the behavior of each system under conditions simulating real application.

**Figure 6:**
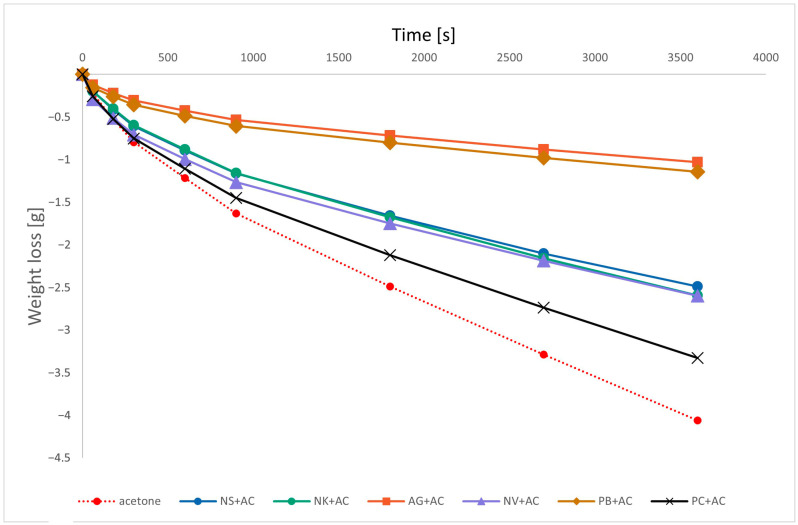
Weight loss (g) curve of acetone-loaded systems vs. neat acetone over time.

All systems reduce the evaporation rate of the free acetone, although to different extents, as reflected by the distinct kinetic profiles. An initial rapid mass-loss phase was observed for all materials, followed by a progressive decrease in the evaporation rate, associated with the release of the more strongly retained solvent within the inner structure (Table 7 and Figure 6).

AG and PB exhibit similar behavior, with both systems showing a rapid decrease in the mass-loss curve (g/min), and an early transition to a quasi-steady evaporation regime. It should be noted, however, that both systems have a limited solvent uptake capacity (~30 mL/100 g for PB; <20% for AG) relative to their total water content.

Both BNC membranes and NV display comparable retention behaviors, with NS showing only a slightly greater deceleration at 60 min.

Conversely, PC exhibited the lowest solvent retention capacity with evaporation behavior closely resembling that of free solvent throughout the experiment.

#### 2.2.2. Diffusion Test

The capillary diffusion test provides insight into the ability of the delivery system to regulate both solvent release and its penetration into the substrate, allowing correlations to be drawn between the fluid retention capacity of the system and the physical properties of the substrate.

Each delivery system, loaded with acetone containing Rodhamine B as a fluorescent marker, was applied to fresco specimens to simulate real cleaning conditions and to account for solvent evaporation from the system during application.

To monitor solvent uptake, the weight of each plaster specimen was measured at regular time intervals during application. The increase in mass, attributed to solvent absorption within the substrate porosity, was recorded and used to construct weight-gain curves as a function of time.

After 10 min and 1 h of application, the specimens were sectioned and examined in cross-section to determine the penetration depth of acetone, by recording Rhodamine B fluorescence under UV illumination.

**Figure 7:**
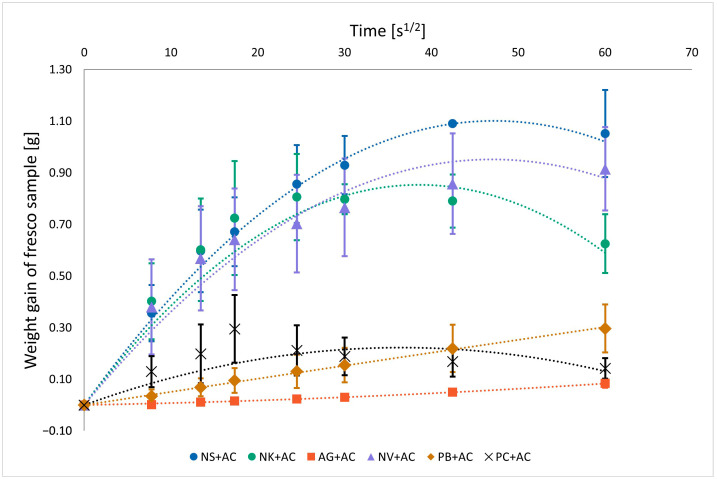
Weight gain (g) of the fresco sample over time, due to the diffusion of the solvent into the porous substrate. Dashed lines show the second-order polynomial fit, calculated using the weighted least squares regression, where the weights are the inverse variance of each data point.

**Figure 8:**
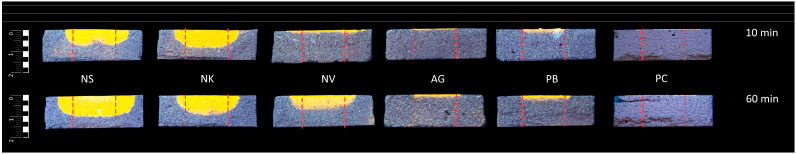
Cross-section of a mortar specimen observed under UV light, showing the penetration depth and lateral diffusion of an acetone–Rhodamine mixture after 10 and 60 min, as visualized by Rhodamine fluorescence. Dashed red lines outline the dimensions of the application area in cross-sectional view.

Overall, the results are consistent with those obtained from the evaporation test (Table 8 and Figure 7). AG and PB proved to be the most retentive systems (0.001 g/min for AG and 0.005 g/min for PB), effectively limiting both in-depth penetration and lateral diffusion (Figure 8).

NV exhibited a rapid solvent release within the first 5 min (0.128 g/min). During this initial phase, the solvent spreads laterally and evaporates quickly from the surface, resulting in limited penetration into the substrate. Subsequently, the slope of the curve decreases markedly (averaging 0.009 g/min), indicating a substantial reduction in solvent transfer as diffusion into the substrate porosity becomes progressively limited.

BNC membranes release a substantial amount of solvent continuously into the substrate. The initial release is immediate and comparable to that of NV (0.134 g/min for NS and 0.144 g/min for NK over the 0–5 min interval). Although BNC membranes provide slightly improved control over lateral diffusion than NV (Figure 8), solvent transfer continues until complete depletion of the membrane reservoir. This behavior may be attributed to the elastic cellulose fibrillar network, which does not provide sufficient rigidity to counterbalance the capillary pressure exerted by the porous mortar. As a result, the solvent uptake by the substrate proceeds as long as the membrane can supply it.

Regarding the PC system, the solvent spreads rapidly and uncontrollably across the specimen surface and evaporates quickly, limiting penetration into the substrate.

Diffusion curves indicate that delivery systems containing neat acetone (NS, NK, PC) exhibit a time-dependent behavior distinct from that of gel-based systems containing both water and solvent (NV, AG, PB). In particular, acetone-only systems show a slope inversion, likely resulting from the combined effects of solvent evaporation from both the system and the mortar surface, together with the progressive cessation of diffusion as the system dries completely.

#### 2.2.3. Residue Release

The persistence of gel residues on treated surfaces may pose a long-term risk of damage or alteration to the original painted materials.

Fresco samples were examined under ultraviolet irradiation after the cleaning treatments to detect fluorescence phenomena associated with modification of the specimen surface after the cleaning treatment with each delivery system.

**Figure 9:**
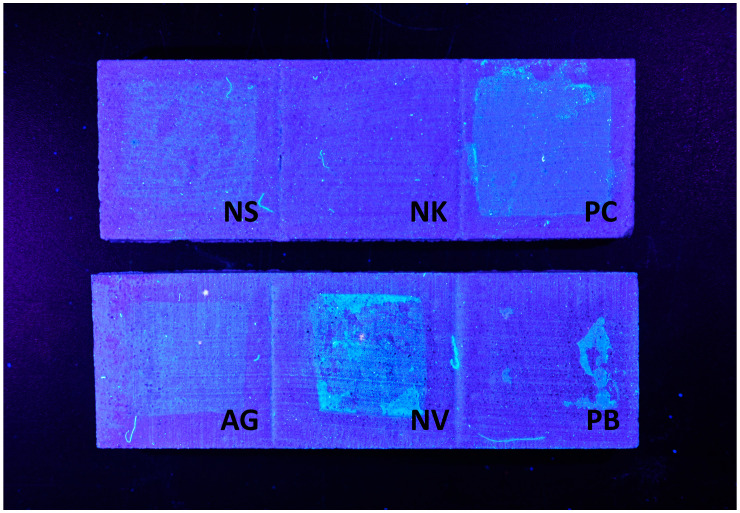
Mortar specimens treated with the selected gels and supporting systems observed using UV light.

UV light observation of samples treated with NV and PB revealed the presence of fluorescence signals of varying intensity (Figure 9).

In particular, the areas treated with PB showed extended regions of strong fluorescence, consistent with the presence of material retained on the surface, in agreement with previous reports describing the tendency of this gel to remain partially adhered to the substrate [71,72,73]. Similarly, the PC-treated areas exhibited visible residual fluorescent regions.

The sample treated with AG displayed a faint and non-uniform yellowish fluorescence. As previously reported in the literature [74,75,76], similar optical effects have been observed even in the absence of chemically detectable gel residues, suggesting that such signals may arise from multiple contributions, including modifications of the substrate or redistribution of gel components during application and removal.

Surfaces treated with NS also exhibited a weak and discontinuous fluorescence signal, whereas the NK-treated areas did not show any detectable fluorescence under the adopted conditions.

As UV fluorescence observations do not allow unequivocal attribution of the observed emission to the possible presence of residues, complementary DRIFT-FTIR analyses were performed on carbonate-based mock-ups not previously coated with Paraloid to avoid spectral interference from the acrylic resin. Samples were collected from areas exhibiting the most pronounced fluorescence after gel application. All measurements were performed on three independent samples, and the complete dataset is provided in the Supplementary Information (Figure S3). The carbonate substrate spectrum was used as a mineral reference baseline to qualitatively evaluate potential additional organic contributions from the applied gel systems, in comparison with both reference spectra and literature data [77,78,79].

The 1200–1000 cm^−1^ region was selected as the most diagnostically useful interval, as other spectral regions were significantly influenced either by residual moisture (O–H stretching) or by the strong carbonate absorption of the substrate (1430–1450 cm^−1^), thus limiting their interpretative value.

The results indicate a good overall overlap between the spectra of untreated samples and those treated with AG, NS, and NK, although a more variable response was observed for NK. Minor variations with respect to the untreated samples were observed for PC and NV, particularly around ~1050 cm^−1^ (C–O and C–O–C vibrations). These features may be tentatively considered compatible with possible polysaccharide-related contributions.

For PB, more pronounced spectral modifications relative to the untreated reference spectrum were observed, consistent with the presence of visible gel residues on the surface. In particular, the region around ~1430 cm^−1^ showed a more intense contribution compared to the untreated sample, potentially attributable to B–O stretching vibrations. This was accompanied by a band around ~1730 cm^−1^, which may correspond to C=O stretching, further supporting the presence of residual material.

Given the detection limits of the technique under the present experimental conditions, more advanced quantitative methodologies would be required to reliably detect and assess potential trace residues.

#### 2.2.4. Cleaning Efficacy Assessment

The aim of the test was to provide a comparative assessment of the performance of different classes of cleaning systems loaded with acetone in removing an acrylic resin from a porous inorganic substrate. Also in this case, the cleaning procedure was limited to the application of the cleaning system onto the paint layer for 5 min, without further clearance. This approach was adopted to exclude operator-induced variability and to specifically observe the interaction of the cleaning systems with the acrylic layer.

The test was not performed with the PC delivery system due to technical limitations. The rapid evaporation of acetone allowed only partial solubilization of the acrylic film, followed by rapid re-hardening. This resulted in the entrapment of cellulose fibers into the swelled layer and prevented complete removal of the poultice, making measurements unfeasible (Figure S4).

**Figure 10:**
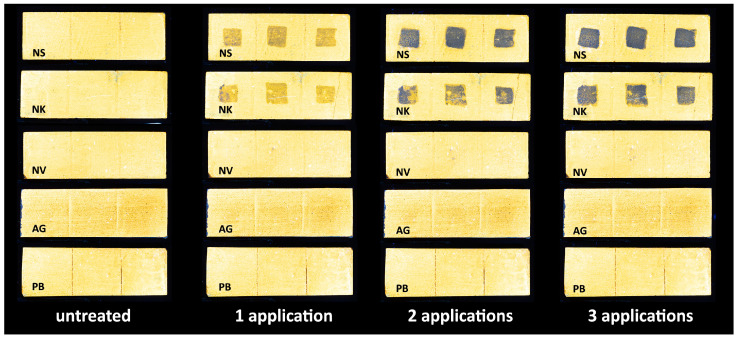
Removal of acrylic resin mixed with Rhodamine as a fluorescent tracer after each application using different delivery systems, observed under UV irradiation.

**Figure 11:**
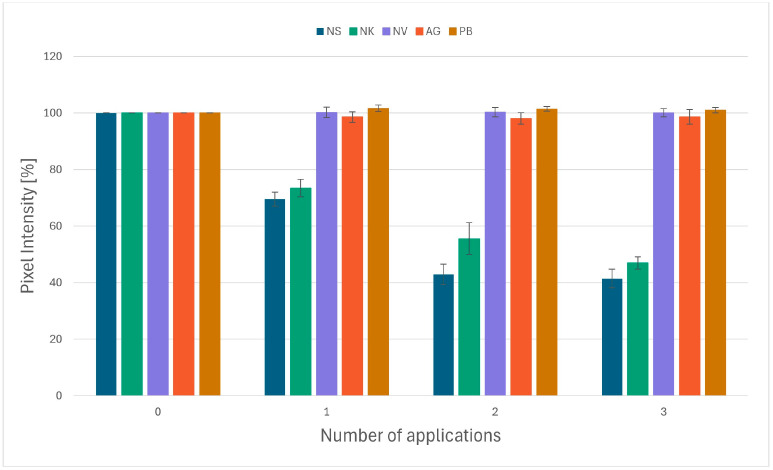
Percentage reduction in pixel intensity within the treated area after each application, calculated from the images shown in Figure 11.

Evaluations were first carried out on mock-up samples coated with Paraloid B72 (CTS S.r.l., Altavilla Vicentina (VI), Italy) labeled with Rodhamine B as fluorescent tracer, using semi-quantitative image analysis in ImageJ version 1.54g (National Institutes of Health, Bethesda, MD, USA). A reduction in pixel fluorescence intensity was used as an indicator of relative removal efficiency. Since minor variations due to surface topography cannot be entirely excluded, the analysis was intended as a comparative evaluation over extended surface areas rather than as an absolute quantification of residual resin. To reduce potential optical artefacts, the results were cross-checked with ATR-FTIR measurements performed on the same specimens. Measurement of the mean gray value (MV) within the selected regions of interest (ROIs) revealed a significant decrease in fluorescence intensity from the first application for both BNC membranes (Figure 10 and Figure 11). NS showed a higher initial removal capacity, with a florescence reduction exceeding 30.5 ± 2.5% after the first application. After three applications, both systems achieved a comparable and uniform reduction in fluorescence (>58.6 ± 3.3% for NS and >53.1 ± 2.2% for NK).

Regarding the other systems, only AG exhibited a measurable—though non-uniform—reduction in MV. This variability can be related to the limited conformability of agar to the rough surface of the specimen.

No significant changes were observed for the remaining systems. A slight increase in fluorescence intensity above initial values observed for NV and PB specimens may be attributed to light-scattering effects, potentially induced by swelling of the coating layer and retention of solvent droplets within the polymer matrix.

**Figure 12:**
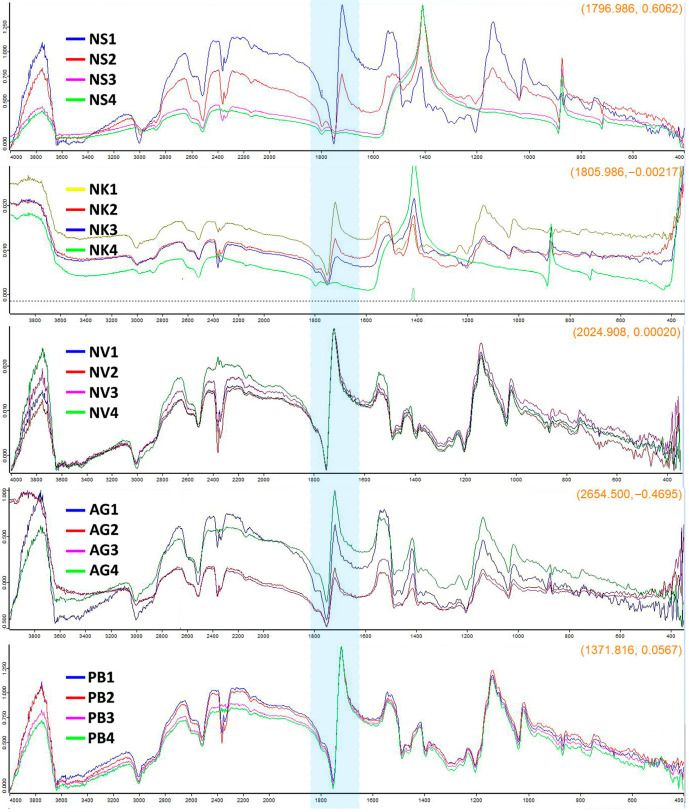
FT-IR spectra acquired in reflection mode; each curve represents the average of three measurements performed on the untreated specimen and after each treatment application.

The results of image analysis were supported by complementary reflectance FT-IR spectroscopy, performed on the same specimens before and after each application cycle, to address potential optical artifacts related to surface morphology changes. In this context, variations in the intensity of the carbonyl band were considered as an indicator of changes in the surface concentration of Paraloid [80]. All reference systems (AG, PB, NV) exhibited essentially unchanged spectral profiles, retaining the characteristic carbonyl (C=O) stretching band at 1730 cm^−1^, with only minimal variations in surface polymer concentration (Figure 12).

Conversely, a reduction in the carbonyl signal was observed for BNC membranes, although with different temporal trends depending on the system. In the case of NK, only slight variations in signal intensity were detected between the first applications (NK2, NK3), while a more pronounced decrease became evident after the third application (NK4) (Figure 12). By contrast, NS exhibited a progressive reduction in the carbonyl signal starting from the first application (NS2), suggesting a more continuous removal process.

**Figure 13:**
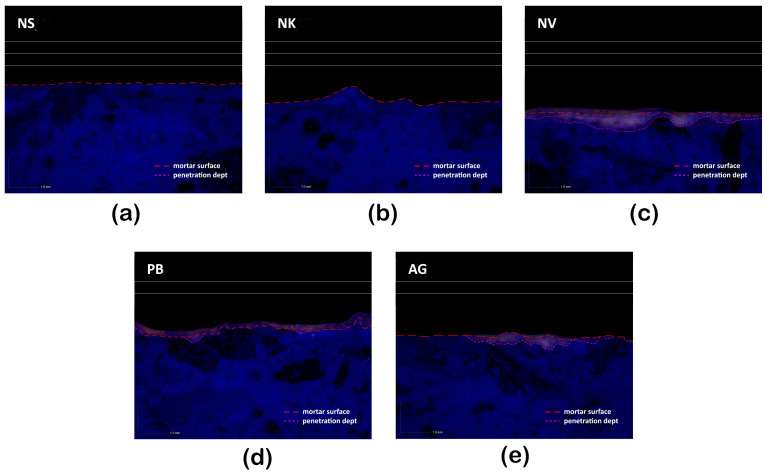
Cross-sections observed under UV light at 50x magnification show mortar specimens after treatment with each delivery system. The most representative images from each set of three samples are presented: NS (**a**), NK (**b**), NV (**c**), PB (**d**), AG (**e**).

**Figure 14:**
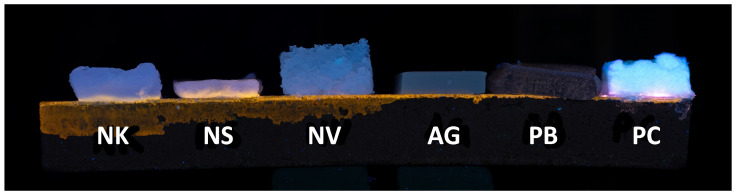
Side view of the delivery systems under UV irradiation after the cleaning treatment. Different fluorescence intensities are observed in the cleaning systems after application to the acrylic resin layer containing Rhodamine B as a fluorescent tracer.

UV observations of cross-sections did not show evidence of solvent-assisted resin penetration into the substrate pore network after treatment with BNC-based systems (Figure 13). Although diffusion tests on mortar confirmed acetone release from both NS and NK into the substrate, no detectable redistribution of the fluorescently labeled resin was observed. This behavior may be interpreted in terms of diffusion-driven transport processes [81,82], in which concentration gradients generated during application govern the redistribution of solvent and solubilized species within the gel network (Figure 14).

However, the absence of detectable fluorescence may also be influenced by significant dilution effects affecting tracer detectability. In the absence of negative control using acetone alone, the underlying mechanisms remain to be fully elucidated. The current dataset does not allow quantitative modeling of solvent and solute diffusion across the substrate–gel interface, limiting a definitive distinction between effective resin removal and redistribution phenomena.

For the other delivery systems, the solubilized resin appeared to penetrate the porous matrix, in some cases reaching depths of up to 0.3 mm, as observed for NV (Figure 13 and Figure S5).

Efficacy tests by FTIR measurements were also carried out in situ on the medieval wall paintings of the Sanctuary of Santa Maria del Piano, confirming the observations previously obtained on laboratory mock-ups.

PC was excluded from the on-site testing due to the macroscopic persistence of residues on the painted surface, including entrapment of cellulose fibers within the swollen polymer matrix, which were clearly visible to the naked eye and raised concerns regarding surface bioreceptivity. PB was also excluded due to the presence of residual polymer films after treatment, likewise macroscopically visible, which exhibited shrinkage phenomena during drying accompanied by localized detachment of the paint layer, representing a potential risk for the painted surface under the tested conditions.

**Figure 15:**
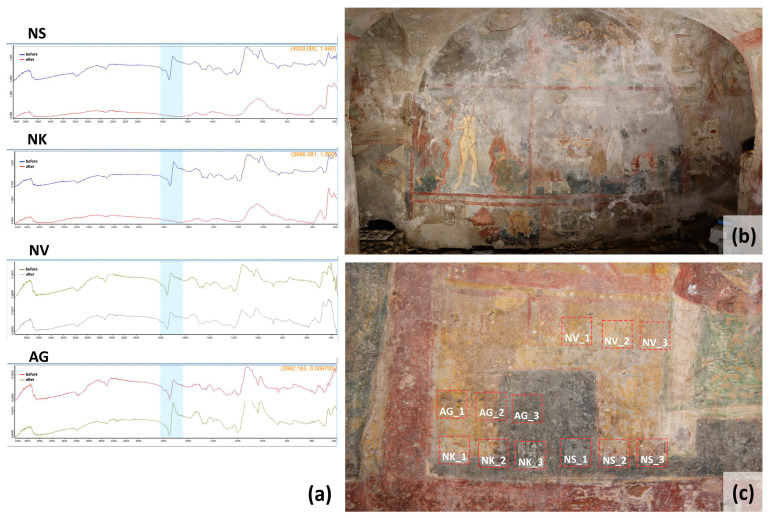
In situ FT-IR measurements: (**a**) FT-IR spectra recorded after a single application of each support system; (**b**) Crypt of Santa Maria del Piano, Ausonia (FR), Italy, south wall; (**c**) detail of the test area.

The treated surfaces showed an almost complete reduction in the characteristic absorption bands of acrylic resin (1739 cm^−1^) after treatment with BNC membranes (Figure 15).

For both membranes, spectra acquired after treatment displayed a stabilized baseline and a clear resolution of the bands associated with calcium carbonate (1400–1450 cm^−1^) and silicates (1100–900 cm^−1^) from the substrate (plaster and pigments). These observations are consistent with a substantial reduction in the surface contribution of the acrylic resin, allowing the spectral features of the underlying substrate to become more evident, even after a single application.

All the remaining systems exhibited only limited spectral changes, as evidenced by the persistence of the characteristic carbonyl band of Paraloid B72. This finding suggests that the treatments did not produce comparable modifications in the surface distribution of the resin under the tested conditions.

## 3. Conclusions

This study presents a preliminary evaluation of bacterial nanocellulose (BNC) membranes as solvent delivery systems for the removal of acrylic resins from mural painting surfaces.

FTIR analysis indicated a comparable chemical composition between laboratory-produced and medical-grade BNCs, suggesting a similar cellulose-based matrix. Conductivity and pH measurements showed overall compatibility with mural substrates, indicating low levels of extractable ionic species under the tested conditions.

Both BNC systems were successfully loaded with acetone via solvent exchange, with optical density measurements indicating negligible release of particulate material and TGA, confirming a substantial reduction in water content after solvent exchange.

FTIR analysis performed to investigate cleaning efficacy showed a more pronounced reduction in the acrylic carbonyl signal after treatment with acetone-loaded BNC compared to the other systems. At the same time, no detectable fluorescent tracer used for resin labeling was observed in cross-sections, even though BNC does not behave as a high-retention gel, as revealed by the solvent diffusion test. The mechanisms underlying these observations remain to be clarified and may involve a combination of solvent-driven diffusion, redistribution phenomena within the porous substrate, and possible interactions of the solubilized resin with the gel matrix.

No detectable evidence of residue release was observed under the adopted experimental conditions; however, quantitative approaches are required to confirm and better assess residual material.

Differences in reproducibility were observed between the two systems, with the medical-grade BNC (NS) showing more homogeneous responses than the laboratory-produced BNC (NK), likely due to inherent variability in raw materials and the production process. Although overall performance was comparable between the two BNC types and similar trends were observed relative to the other delivery systems tested, this difference in reproducibility may represent a relevant factor for their practical selection in conservation applications.

Overall, the study provides a structured methodological framework for the evaluation of BNC-based delivery systems, integrating physicochemical, mechanical, and spectroscopic analyses under application-oriented conditions.

Further research is needed to clarify residue behavior, particularly in relation to aging effects and potential substrate bioreceptivity, as well as to better understand the relationship between BNC microstructure and its solvent transport and mechanical properties.

## 4. Materials and Methods

### 4.1. Bacterial Nanocellulose Membranes

Commercial medical grade BNC membrane Suprasorb X^®^ (Lohmann & Rauscher GmbH & Co. KG, Rengsdorf, Germany) was chosen, based on literature data [83], for its high performance and elevated grade of purity.

Kombucha BNC membranes were obtained by selecting the highest-yielding medium composition and growth parameters, based on procedures reported in the literature and in preliminary trials [24,34,35,47,54,56,84,85,86,87]. Black tea leaves (10 g/L; Ceylan Tea, Sri Lanka, Epel Tea, Sakito & Kuki S.L.,Arrasate (Gipuzkoa), Spain) were brewed in distilled water at 90 °C for 10 min; 100 g/L of commercial food grade sugar (Sadam, Eridania Italia S.p.A., Bologna, Italy) was added and a liquid starter (20% *v*/*v*), provided by home-made batch, was added after cooling. The pH was adjusted to 3.5 by adding glacial acetic acid (Carlo Erba Reagents S.r.l., Cornaredo (MI), Italy). Next, 773 mL glass jars were filled with 600 mL of culture medium, loosely covered with plastic lids and placed in a thermostatic bath maintained at 28 ± 3 °C. The sweetened tea broth was left to ferment in the dark for 8–10 days. After this period, 5–8 mm thick pellicles were harvested and purified by immersion in 0.5 M sodium hydroxide solution at 80 °C for 60 min and autoclaved at 121 °C for 30 min.

Kombucha-derived BNC membranes were immersed in deionized water baths for 10 days, with daily replacement of the water, until high clarity of the washing solution and neutral pH values were achieved.

Suprasorb X^®^ membranes were used as supplied and were rinsed in deionized water for 2 days prior to use.

### 4.2. Reference Delivery Systems and Gels

The reference systems were prepared according to the instructions provided in the technical data sheets or based on literature reports.

- Agar–agar (AgarArt^®^, CTS S.r.l., Altavilla Vicentina (VI), Italy) powder (3% *w*/*v*) was dispersed in demineralized water and heated to 95 °C under stirring until complete dissolution. Acetone (30% *v*/*v*) was added at 40 ± 5 °C prior to gel setting.
- NEVEK^®^ (CTS S.r.l., Altavilla Vicentina (VI), Italy) was loaded with solvent (30% *v*/*w*) to obtain a non-dripping consistency suitable for application.
- Poly(vinyl alcohol) (PVA molecular weight 50,000, hydrolysis 87–89%, Antares S.r.l., San Lazzaro di Savena, (BO), Italy) was dissolved in demineralized water (8% *w*/*v*) at 95 °C under stirring until a clear solution was obtained. A borax aqueous solution (8% *w*/*v*) was added to the PVA solution at a 1:4 volume ratio to induce gelation [88]. Before borax addition, acetone was incorporated into the aqueous poly(vinyl alcohol) (PVA) solution at a ratio of 30% relative to the final PVA–borax mixture.
- Paper pulp (Arbocel^®^ BWW40, JRS PHARMA GmbH & Co. KG, Rosenberg, Germany) was loaded with appropriate amounts of acetone (paper pulp/acetone ratio: 1/2 *w*/*w*) by absorption and manual mixing.

### 4.3. Fresco Specimens

Fresco mock-ups were prepared using lime-based mortars with a binder-to-aggregate ratio of 2:1 (*v*/*v*). A 24-month-aged lime putty (Cepro 550, Cromology Italia Spa, Porcari (LU), Italy) was used as the binder.

The aggregate mixture consisted of two parts river sand (Ø ≤ 0.25 mm, Bacchi S.p.A., Boretto, RE, Italy), one part calcarenite powder (Ø < 0.25 mm) (Tufina powder from Montescaglioso (MT), Italy) and one part pumice powder (0.15 < Ø < 0.18 mm), (Antares S.r.l., San Lazzaro di Savena, BO, Italy).

A white limewash layer of Bianco San Giovanni (slaked lime/water ratio 5:1, *v*/*v*) was applied onto the moist plaster before completion of the carbonation process. The pigment-to-binder ratio was 1:4.

Only for the cleaning efficacy tests, an acrylic resin solution (Paraloid B72, 20% *w*/*v* in acetone) containing Rhodamine dye as a fluorescent tracer (0.08% *w*/*v*) was homogeneously applied to the fresco samples.

### 4.4. Characterization of BNC Membranes

#### 4.4.1. Chemical Compatibility Assessment for Conservation Applications

To compare the laboratory production and purification process of NK with industrially produced NS, ATR-FTIR measurements were performed on dry specimens of both types [54].

FTIR measurements were performed by using a Nicolet iN10 MX Microscope (Thermo Fisher Scientific, Waltham, MA, USA) equipped with a MCT/A detector cooled with liquid nitrogen in a measurement range of 4000–675 cm^−1^. Spectral collection was made in transmission mode, accumulating 16 scans in 3.07 s at a resolution of 8 cm^−1^, using Omnic Picta software version 1.9.22 (Thermo Fisher Scientific, Waltham, MA, USA).

Additionally, pH and electrical conductivity were measured to assess the compatibility of BNC membranes with original materials. Measurements were carried out in triplicate on 50 mL of demineralized water after immersion of BNC membrane samples (20 × 20 mm^2^) for 24 h under stirring conditions. Results are reported as mean values.

#### 4.4.2. Water Holding Capacity (WHC) and Water Release Rate (WRR)

To determine the Water Holding Capacity (WHC) of the two BNC membranes, 20 × 20 mm^2^ samples were swollen to equilibrium in demineralized water and gently shaken to remove excess surface water.

To ensure identical starting conditions, the laboratory-produced BNC was oven-dried to achieve the same percentage hydration level as the packaged commercial type.

The swollen weight (*Ww*) was recorded, after which the samples were dried in an oven (Universal Oven UN30, Memmert GmbH & Co. KG, Schwabach, Germany) at 60 °C for 24 h to obtain the dry weight (*Wd*). WHC was then calculated using the following formula [32,35,63]:

(1) WHC=(Ww−Wd(g))/(Wd(g))

WRR was determined by monitoring mass loss (%) of wet BNC membranes under controlled conditions (40 ± 3 °C, oven, flap 100%). The Water Release Rate (WRR), expressed as %/min, was calculated from the mass loss over time, normalized to the initial mass of the system. Specifically, WRR was determined as the slope of the mass loss curve (Δ*m*/Δ*t*), expressed as a percentage of the initial mass (m_0_), according to the following equation [32,35,63]:

(2) $$WRR\;(\%/min)=1/m_{0}\;\cdot \Delta m/\Delta t\times 100$$

Three-dimensional variations were also documented after 1 h and 24 h immersion in water. All the experiments were conducted in triplicate and results reported as mean values.

#### 4.4.3. Solvent-Loading Assessment

Hydrated NS and NK membranes underwent a stepwise solvent-exchange protocol, transitioning from water to ethanol to acetone. The exchange process was carried out by steps through sequential 30-min immersions in solvent mixtures with an increasing content of less polar solvent (25/75%, 50/50%, 75/25%) [56,89], as shown in Table 9.

The effectiveness of the loading method was investigated through the use of a thermogravimetric analyzer coupled with a differential scanning calorimetry TGA/DSC 3+ STARe System (Mettler Toledo International Inc., Columbus, OH, USA). Approximately 30 mg of each sample was placed in a 150 μL Al_2_O_3_ crucible and heated up to 200 °C at a constant heating rate of 10 C°/min under a nitrogen atmosphere and a flow rate of 50 mL/min. Several parameters were directly computed by analyzing the weight loss (TG) curves and their respective first-order derivatives (DTG) to compare the evaporation profiles.

#### 4.4.4. Tensile Testing

The mechanical properties of the BC hydrogel were evaluated by tensile testing using an AML Z10 double-column universal testing machine (UTM) equipped with a 1 kN load cell (AML Instruments Limited Ltd, Lincoln, Lincolnshire, UK).

In the present study, specimens measuring 45 ± 3 mm in length (between clamping lines) and 16 ± 2 mm in width were used [90,91].

Both NS and NK specimens were tested after loading with water or acetone. Samples were equilibrated to a solvent content of 15% by mass prior to testing to ensure proper clamping between the grips. Tensile tests were performed at a crosshead speed of 1.0 mm min^−1^. Due to the compliant nature of the BNC hydrogel, a preload of 0.1 N was applied. For each condition, average values were calculated from three independent specimens.

Mechanical behavior was evaluated in terms of maximum load (Fmax), ultimate tensile strength (*σ*), and elongation at break (*ε*), calculated according to the following equations:

(3) σ=Fmax/A,

where *Fmax* is the maximum load recorded during the tensile test and *A* is the initial cross-sectional area of the specimen.

(4) $$\varepsilon =(L-L_{0})/L,$$

where *L*_0_ is the initial gauge length of the specimen and *L* is the length at break.

#### 4.4.5. Mechanical Integrity in Solvents

After solvent exchange, NS and NK samples (5 × 5 × 5 mm^3^) were immersed in 5 mL of water, ethanol, or acetone for 48 h and subsequently subjected to reciprocating shaking for 2 h to promote the release of loosely bound residues into the surrounding medium. The immersion solutions were then collected for optical density analysis.

Optical density was evaluated by visible-range spectrophotometry (400–750 nm) using a UV-1601 spectrophotometer (Shimadzu Corporation, Kyoto, Japan). Measurements were performed in 1 cm path-length cuvettes containing 4 mL of immersion solution. For each solvent, the corresponding pure solvent was used as a blank. The recovered immersion media were analyzed against their respective blanks.

Any increase in absorbance relative to the solvent blank was interpreted as indicative of residual material released from the BNC matrix. All experiments were performed in triplicate using independent samples.

### 4.5. Comparative Tests

#### 4.5.1. Solvent Retention Test

Six disk-shaped samples (8 cm in diameter and 7 ± 1 mm thickness), one for each product, were prepared and loaded with acetone, following the procedures described above. The samples were placed in an oven maintained at 25 ± 3 °C with its exhaust flap fully open. Weight loss for each specimen was recorded over time and the weight/time loss curve was plotted.

The evaporation flux rate (*J*) for each time interval was calculated utilizing the following equation:

(5) J=Δw/(A·Δt),

The relative evaporation rate (ῡ%) of acetone-loaded systems with respect to neat acetone over 60 min was calculated as:

(6) ῡ% =ῡ/ῡ0× 100,

where ῡ is the system evaporation rate and ῡ0 is the neat acetone evaporation rate.

#### 4.5.2. Diffusion Test

Diffusion tests were performed by applying acetone loaded systems (20 × 20 × 7 ± 1 mm^3^) onto fresco specimens for 1 h. The mass variation in the sample was monitored throughout the experiment and the diffusion rate (ῡ) of the solvent into the specimen was calculated at each time interval, measuring the weight gain of the fresco sample due to the solvent diffusion inside the porosity of the specimen. The diffusion rate was determined according to the following formula:

(7) ῡ = Δw/Δt,

Tests were performed in triplicate and results are reported as mean values.

Rhodamine B (0.1% *w*/*v*) was added to acetone to enable the visual assessment of horizontal diffusion and penetration depth after 10 and 60 min’ applications, via cross-sectional observation of the sample under UV irradiation. The resulting fluorescence in the visible spectrum was recorded using a digital camera.

The Rhodamine solution was visually inspected to ensure complete dissolution, resulting in a homogeneous phase with no observable precipitation or aggregates.

#### 4.5.3. Residue Release

Surface modification induced by treatments was investigated by visual inspection of mortar samples under UV irradiation (λ = 360 nm) after the treatment. Pads of each supporting system (40 × 40 mm^2^) were applied onto the surface and removed after 5 min. No additional clearance of the surface with swabs was performed. The fluorescence induced by UV excitation was documented using a digital camera.

To further investigate the origin of the observed fluorescence, additional analyses were carried out by FT-IR spectroscopy on specifically prepared carbonate-based samples, selected to minimize spectral interference from the substrate. FT-IR spectra were acquired using a Nicolet Avatar 360 spectrometer (Thermo Fisher Scientific, Waltham, MA, USA) equipped with (DTGS) detector, Michelson interferometer in DRIFT mode, in the 4000–400 cm^−1^ spectral range, with a resolution of 4 cm^−1^. Each spectrum represents an average of 128 scans.

#### 4.5.4. Cleaning Efficacy Assessment

To compare cleaning efficacy of BNC membranes with reference products a semiquantitative image analysis protocol was developed using ImageJ.

An acrylic resin solution (Paraloid B72) in acetone (20% *w*/*v*) incorporating Rhodamine dye marker, was applied homogeneously on fresco samples. Images of each mortar sample acquired prior to the application of the acrylic layer were used as background references for image analysis.

Pads of each supporting system (20 × 20 mm^2^) loaded with acetone were applied onto the surface and removed after 5 min. This procedure was repeated three times. No additional cleaning steps, such as surface refinement with swabs, were performed.

For NV and PC, a sheet of Japanese paper was interposed between the pad and the surface to minimize residue deposition and facilitate removal.

UV fluorescence images (λ = 360 nm) were acquired before treatment and after each application. All images were digitally aligned and processed in ImageJ. The background signal—determined from the untreated specimen—was subtracted. The mean reflectance of the treated areas was then measured within a selected “Region of Interest” (ROI), corresponding to the treatment area, before and after each application. The removal efficiency was estimated as the percentage decrease in pixel intensity within the selected ROI.

To ensure data reliability, all applications were performed in triplicate.

After measuring, the specimens were cut and cross-sections observed by digital microscope at 50× under visible and UV light, to detect possible solubilized acrylic resin penetration into the substrate.

FTIR measurements were carried out to support the results obtained through quantitative image analysis on mock-up specimens. Analyses were performed on untreated samples and after each of the three applications of the cleaning system, to monitor the progressive removal of the acrylic film.

FT-IR spectra were acquired using a Bruker ALPHA II Compact portable spectrometer (Bruker Optics GmbH, Ettlingen, Germany) equipped with an external reflection module, in the 4000–400 cm^−1^ spectral range, with a resolution of 4 cm^−1^. Each spectrum represents an average of 16 scans. Background spectra were collected prior to each measurement using a reference mirror. Spectral processing was conducted with OPUS version 8.1 (Bruker Optics GmbH, Ettlingen, Germany).

The same measurement protocol was applied in situ, collecting spectra before and after treatment with each supporting system.

All measurements were performed in triplicate.

## Figures and Tables

**Table 1:** Delivery systems comparatively investigated in this study.

Abbreviation	Commercial Name	Composition	Category
NS	Suprasorb X^®^ (Lohmann & Rauscher GmbH & Co. KG, Rengsdorf, Germany)	BNC	Commercial medical-grade BNC membrane
NK	Kombucha Scoby	BNC	Laboratory-produced BNC membrane
AG	Agar Art^®^ (CTS S.r.l., Altavilla Vicentina (VI), Italy)	Agarose and agaropectine	Rigid hydrogel
NV	Nevek^®^ (CTS S.r.l., Altavilla Vicentina (VI), Italy)	Agarose and agaropectine	Homogenized grounded hydrogel
PB	PVA-borax gel (Antares S.r.l., San Lazzaro di Savena (BO), Italy)	Poly(vinyl alcohol) + sodium borate	Highly viscous polymeric dispersion (HVPD)
PC	Arbocel^®^ BWW40 (JRS PHARMA GmbH & Co. KG, Rosenberg, Germany)	Cellulose fibers	Paper pulp

**Table 2:** Experimental setup.

Experimental Phase	Property Investigated	Test Performed	Products Tested
(1) Characterization of BNC membranes	Chemical compatibility for conservation application	pH; conductivity; ATR-FTIR	NS; NK
	Water Holding Capacity	gravimetric measurement	NS; NK
	Water Release Rate	gravimetric measurement until complete drying	NS; NK
	Solvent loading	thermogravimetric and differential scanning calorimetry (TG–DSC)	NS; NK
	Tensile properties	tensile test	NS; NK
	Integrity during solvent loading	optical density measurement	NS; NK
(2) Comparative assessment against reference materials	Solvent retention	gravimetric measurement	NS; NK; NV; AG; PB; PC
	Diffusion test	gravimetric measurement; cross-section observation with Rhodamine marker	NS; NK; NV; AG; PB; PC
	Residue release	ultraviolet-induced fluorescence imaging analysis; DRIFT-FTIR	NS; NK; NV; AG; PB; PC
	Cleaning efficacy on mock ups	quantitative image analysis with Rhodamine marker; FTIR reflection mode	NS; NK; NV; AG; PB
	Cleaning efficacy on site	FTIR reflection mode	NS; NK; NV; AG

**Table 3:** pH and conductivity values of the soaking water for NS and NK BNC membranes.

Sample	pH	Conductivity (μS/cm)
Soaking water	6.3 ± 0.15	2.9 ± 0.1
NS	6.3 ± 0.05	18.3 ± 0.2
NK	6.4 ± 0.10	14.3 ± 0.5

**Table 4:** WRR and WHC values of NS and NK membranes.

BNC	WHC (g H_2_O/g BNC)	WRR (%/min)
NS	151.94 ± 1.77	−0.227 ± 0.010
NK	173.12 ± 23.75	−0.243 ± 0.019

**Table 5:** Dimensional changes in NS and NK membranes after 24 h water immersion.

Samples	Immersion Time	In-Plane Dimensional Variation (mm)		Vertical Swelling (mm)
		x	y	z
NS	1 h	0	0.03 ± 0.06	4 ± 0.3
	24 h	0	0.07 ± 0.12	6 ± 0.2
NK	1 h	0.07 ± 0.06	0.03 ± 0.06	1.9 ± 0.2
	24 h	0.03 ± 0.06	−0.03 ± 0.06	5.2 ± 0.4

**Table 6:** Tensile properties of NS and NK specimens loaded with water and acetone. *Fmax*, *σ*, and *ε*% denote tensile strength, tensile stress and strain, respectively.

Sample	Loaded Solvent	*Fmax* (N)	*σ* (MPa)	*ε*%
NK	Water	12.38 ± 1.4	0.76 ± 0.14	22.05 ± 0.54
	Acetone	49.14 ± 16.43	2.97 ± 1.06	4.69 ± 2.76
NS	Water	13.09 ± 1.02	0.92 ± 0.09	19.99 ± 3.90
	Acetone	28.99 ± 6.16	1.63 ± 0.39	10.12 ± 1.85

**Table 7:** Evaporation behavior of acetone-loaded systems vs. neat acetone (AC) over time.

Sample	*J*_0–1_	*J*_1–3_	*J*_3–5_	*J*_5–10_	*J*_10–15_	*J*_15–30_	*J*_30–45_	*J*_45–60_	ῡ (mg/cm^2^∙min)	Δm Tot (g)	ῡ %
AC	44.285	30.787	26.151	16.783	16.520	11.352	10.573	10.256	1.3457	4.0584	100.0%
PC + AC	51.288	26.290	22.789	14.119	13.626	0.8883	0.8171	0.7862	1.1032	3.3271	82.0%
NS + AC	39.053	21.755	19.049	11.324	10.791	0.6564	0.5876	0.5143	0.8249	2.4880	61.3%
NK + AC	39.411	20.332	19.158	11.364	11.033	0.6866	0.6369	0.5804	0.8599	2.5935	63.9%
AG + AC	24.669	0.9519	0.8614	0.4723	0.4393	0.2383	0.2186	0.2001	0.3418	1.0308	25.4%
NV + AC	58.290	21.794	19.427	11.455	10.731	0.6415	0.5768	0.5467	0.8607	2.5958	64.0%
PB + AC	30.856	10.673	0.9291	0.5181	0.4751	0.2589	0.2345	0.2200	0.3791	1.1433	28.2%

*J* = evaporation flux at each time interval, ῡ = mean evaporation rate, Δm tot = total evaporation mass, ῡ% = relative evaporation rate.

**Table 8:** Diffusion rate (ῡ = Δ*w*/Δ*t*) of acetone-loaded systems into the fresco samples at each time interval, and total diffused solvent mass (Δm_tot_).

Sample	ῡ_0–1_ (g/min)	ῡ_1–3_ (g/min)	ῡ_3–5_ (g/min)	ῡ_5–10_ (g/min)	ῡ_10–15_ (g/min)	ῡ_15–30_ (g/min)	ῡ_30–60_ (g/min)	Δm_tot_ (g)
NS + AC	0.3560 ± 0.1093 *	0.1206 ± 0.0969	0.0369 ± 0.1042	0.0370 ± 0.0403	0.0147 ± 0.0377	0.0107 ± 0.0075	−0.0013 ± 0.0056	1.0902 ± 0.0054
NK + AC	0.4023 ± 0.1471	0.0998 ± 0.1235	0.0613 ± 0.1484	0.0164 ± 0.0554	−0.0018 ± 0.0353	−0.0005 ± 0.0079	−0.0055 ± 0.0051	0.8063 ± 0.1669
AG + AC	0.0015 ± 0.0069	0.0049 ± 0.0042	0.0020 ± 0.0036	0.0016 ± 0.0014	0.0013 ± 0.0011	0.0013 ± 0.0004	0.0011 ± 0.0006	0.0837 ± 0.0167
NV + AC	0.3805 ± 0.1837	0.0942 ± 0.1365	0.0365 ± 0.1409	0.0123 ± 0.0546	0.0125 ± 0.0535	0.0061 ± 0.0181	0.0019 ± 0.0084	0.9148 ± 0.1615
PB + AC	0.0342 ± 0.0260	0.0170 ± 0.0217	0.0133 ± 0.0297	0.0071 ± 0.0161	0.0049 ± 0.0185	0.0043 ± 0.0076	0.0026 ± 0.0044	0.2968 ± 0.0931
PC + AC	0.1295 ± 0.0607	0.0345 ± 0.0868	0.0484 ± 0.0868	−0.0167 ± 0.0326	−0.0046 ± 0.0243	−0.0013 ± 0.0062	−0.0009 ± 0.0024	0.2951 ± 0.1310

* Standard deviation propagated: SD_v_ = SD_Δm_/Δ*t*.

**Table 9:** Solvent loading steps of BNC samples.

Loading Process	Sample Label	H_2_O %	H_2_O/Ethanol %			Ethanol %	Ethanol/Acetone %			Acetone %
		100	75/25	50/50	25/75	100	75/25	50/50	25/75	100
Solvent exchange	NS_H_2_O	x								
	NS_AL	x	x	x	x	x				
	NS_AC	x	x	x	x	x	x	x	x	x
	NK_H_2_O	x								
	NK_AL	x	x	x	x	x				
	NK_AC	x	x	x	x	x	x	x	x	x

## Data Availability

The original contributions presented in this study are included in the article/Supplementary Material. Further inquiries can be directed to the corresponding author.

## Supplementary Materials

The following supporting information can be downloaded at: https://www.mdpi.com/article/10.3390/gels12060551/s1, Figure S1. Vertical swelling and in-plane dimensional changes of BNC membrane at 0 h, 1 h and 24 h: (**a**) NK1, (**b**) NK2, (**c**) NK3, (**d**) NS1, (**e**) NS2, (**f**) NS3; Figure S2. DSC, TGA and DTG curves of both BNC membranes (NS and NK) loaded with water (**a**,**d**), ethanol (**b**,**e**) and acetone (**c**,**f**); Figure S3: DRIFT-FTIR spectra of mortar samples after treatment with different cleaning systems. Spectra were acquired on three independent samples and are displayed with vertical offsets for clarity, in comparison with the untreated reference sample; Figure S4. Mortar sample with a superimposed acrylic layer containing Rhodamine tracer, observed under visible light (**a**) and UV light (**b**) after PC+ acetone application. The image shows the presence of cellulose powder particles entrapped within the swollen acrylic film; Figure S5. Cross-sections of fresco specimens at 50× magnification after cleaning. The acrylic layer, marked with a Rhodamine tracer, was removed using each delivery system in triplicate: (**a**) NS1, NS2, NS3; (**b**) NK1, NK2, NK3; (**c**) NV1, NV2, NV3; (**d**) AG1, AG2, AG3; (**e**) PB1, PB2, PB3. Table S1. Thermal parameters of solvent-loaded BNC membranes expressed as mean values of three independent samples.
