# Supplementary material for: Preliminary Assessment of BNC Membranes as Solvent Delivery Systems for the Cleaning of Mural Paintings: Comparison with Traditional Gel Systems
Source: Gels. 2026 Jun 19;12(6):551. doi: 10.3390/gels12060551 (PMC13298414; doi:10.3390/gels12060551)
Supplement: Supplementary file 1 [file gels-12-00551-s001.zip › gels-4320128-supplementary.pdf]

gels

Supplementary materials

Assessment of BNC membranes as Solvent Delivery System for the Cleaning of Artworks: Comparison with Traditional Gel Systems

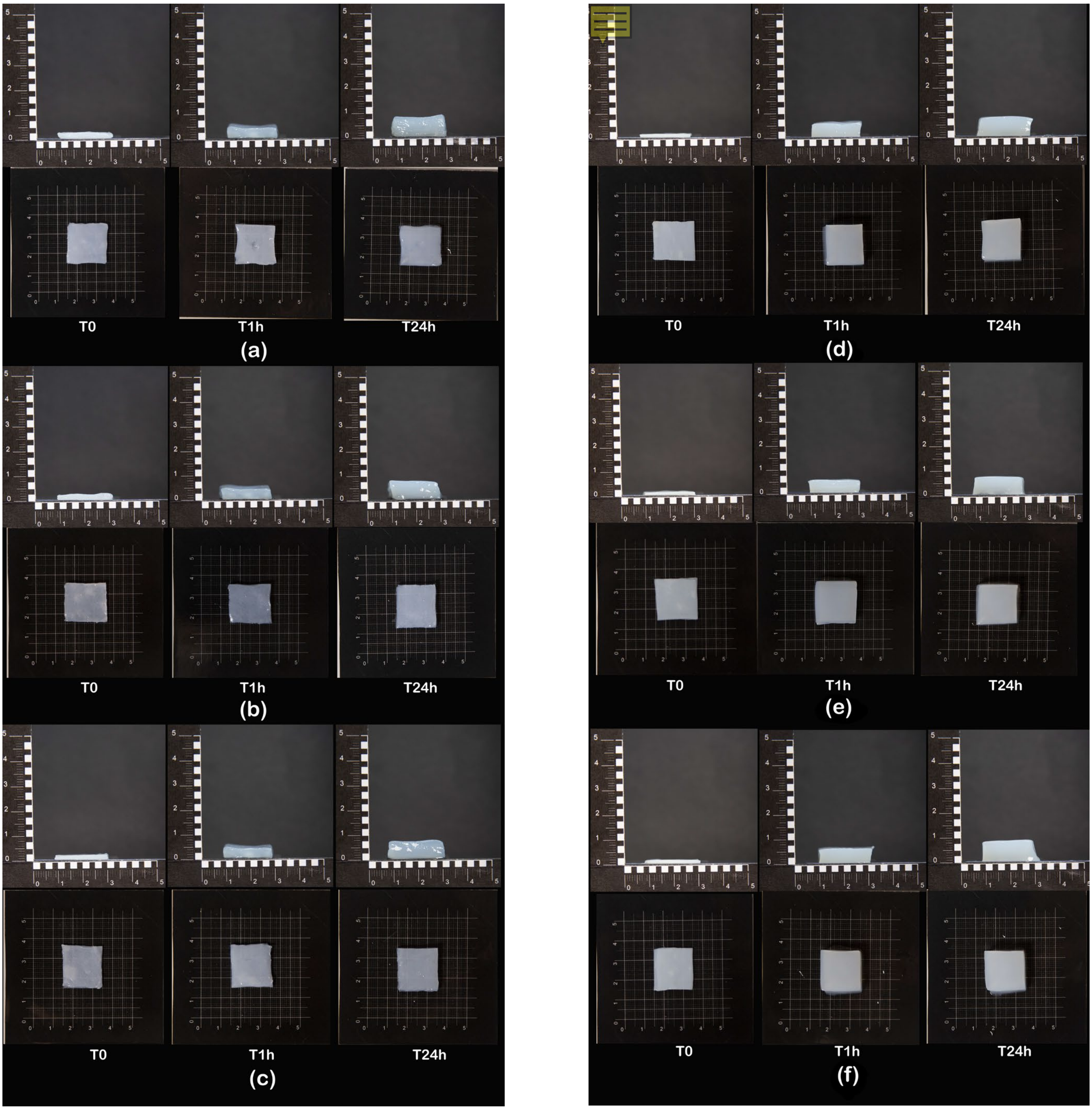

Figure S1. Vertical swelling and in-plane dimensional changes of BNC membrane at 0h, 1h and 24h: (a) NK1, (b) NK2, (c) NK3, (d) NS1, (e) NS2, (f) NS3.

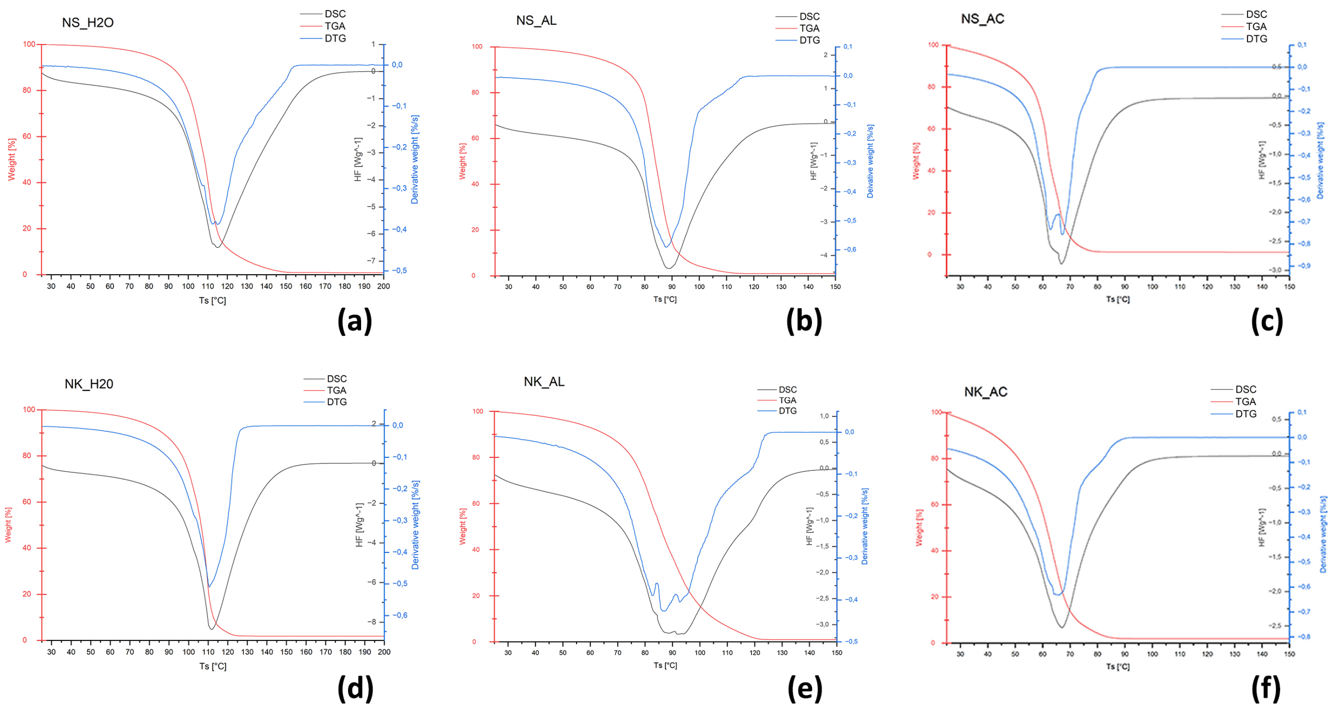

Figure S2. DSC, TGA and DTG curves of both BNC membranes (NS and NK) loaded with water (a,d), ethanol (b,e) and acetone (c,f).

Table S1. Thermal parameters of solvent-loaded BNC membranes expressed as mean values of three independent samples.

Sample	Tonset (°C)	Tpeak (°C)	Tendset (°C)
NS_H_2O	103.4 ± 2.0	114.0 ± 0.9	152.4 ± 9.0
NK_H_2O	96.4 ± 7.3	114.3 ± 4.4	138.9 ± 11.9
NS_AL	79.9 ± 4.5*	89.0 ± 1.1	119.7 ± 2.7
NK_AL	70.4 ± 2.3	89.5 ± 5.0	123.4 ± 5.9
NS_AC	54.3 ± 6.5	67.4 ± 3.1	87.1 ± 1.4*
NK_AC	54.6 ± 8.0	65.8 ± 2.5	93.3 ± 1.5

* indicate statistically significant differences between NS and NK ($p < 0.05$).

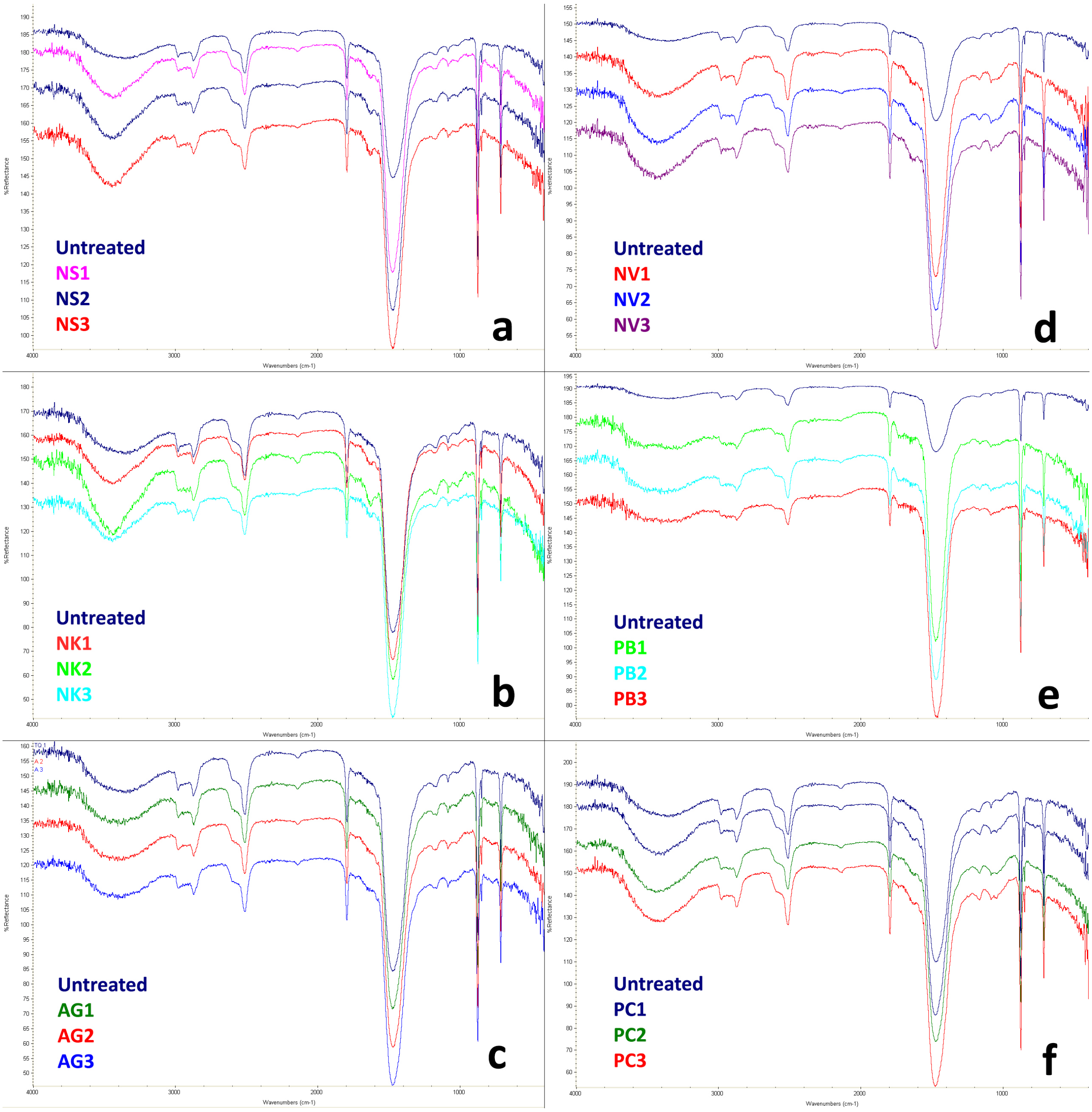

Figure S3: DRIFT-FTIR spectra of mortar samples after treatment with different cleaning systems. Spectra were acquired on three independent samples and are displayed with vertical offsets for clarity, in comparison with the untreated reference sample.

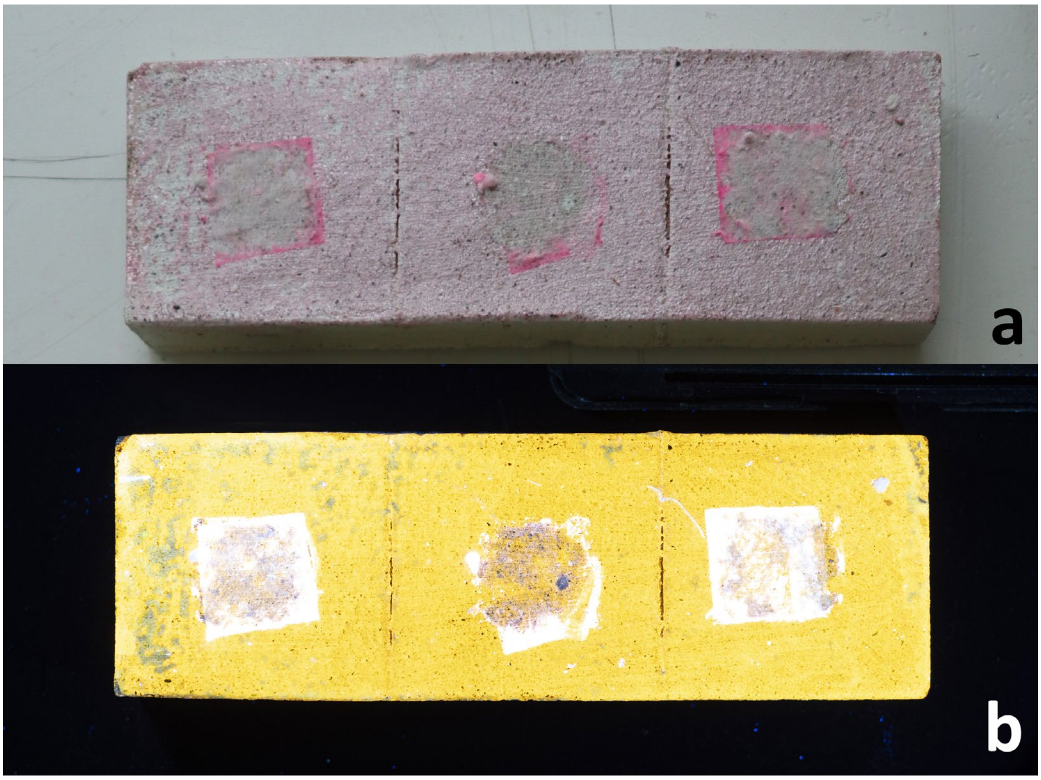

Figure S4. Mortar sample with a superimposed acrylic layer containing Rhodamine tracer, observed under visible light (a) and UV light (b) after PC+ acetone application. The image shows the presence of cellulose powder particles entrapped within the swollen acrylic film

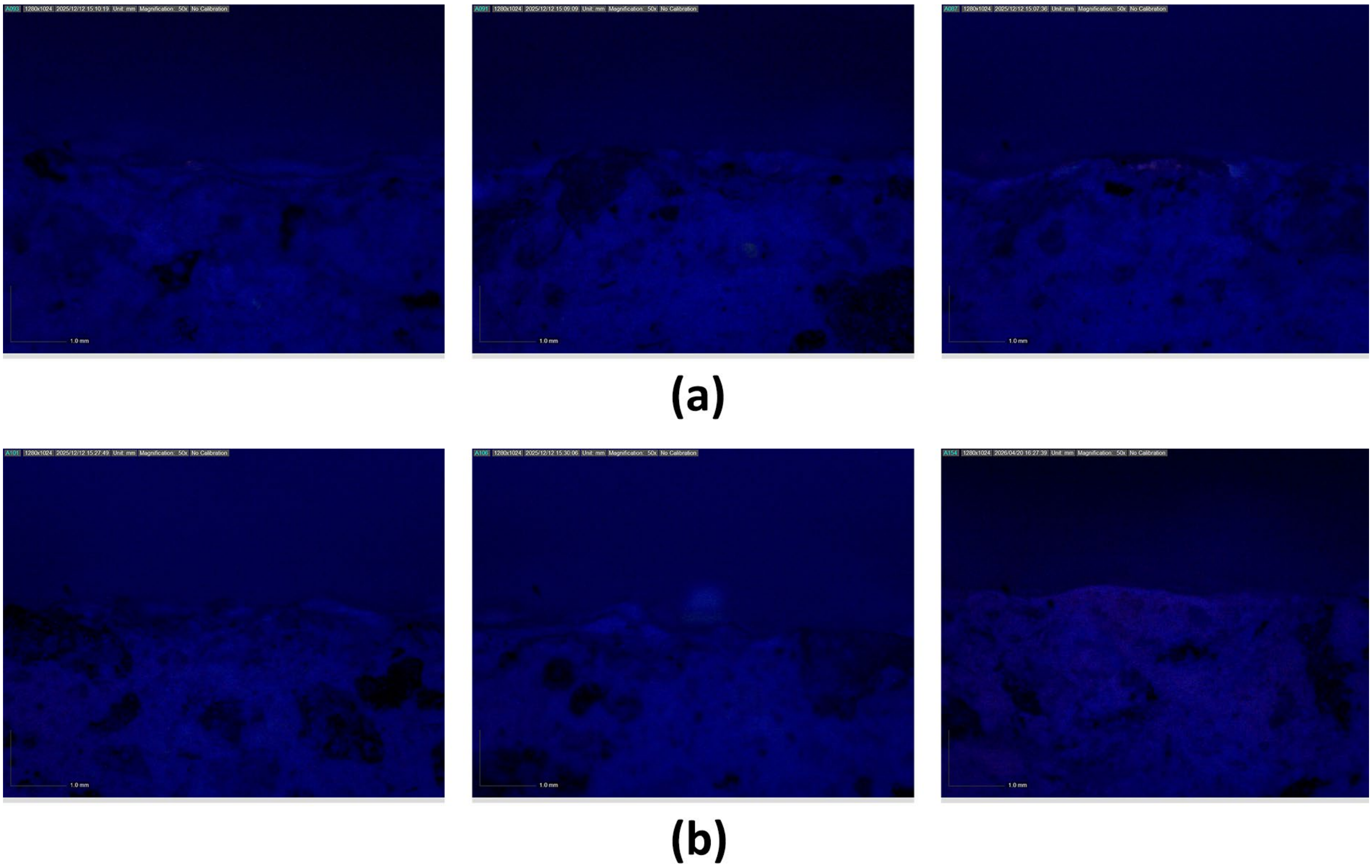

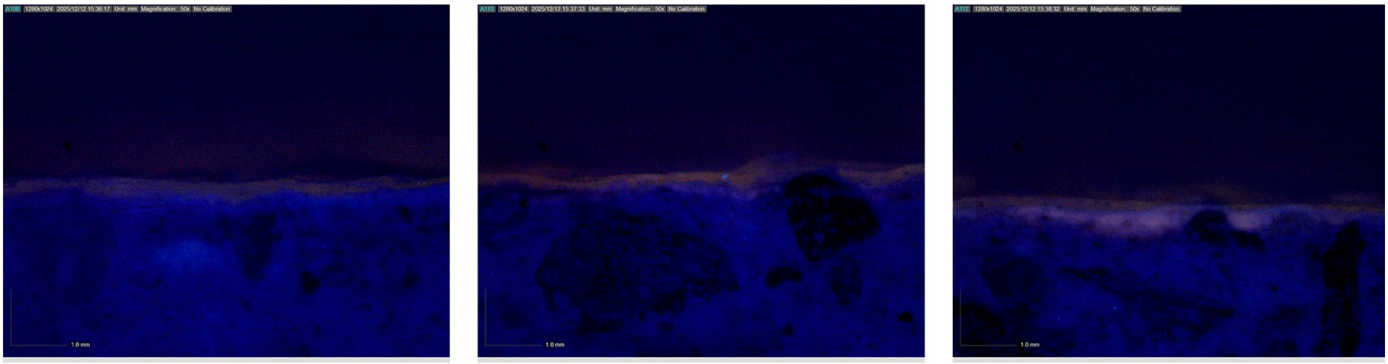

(c)

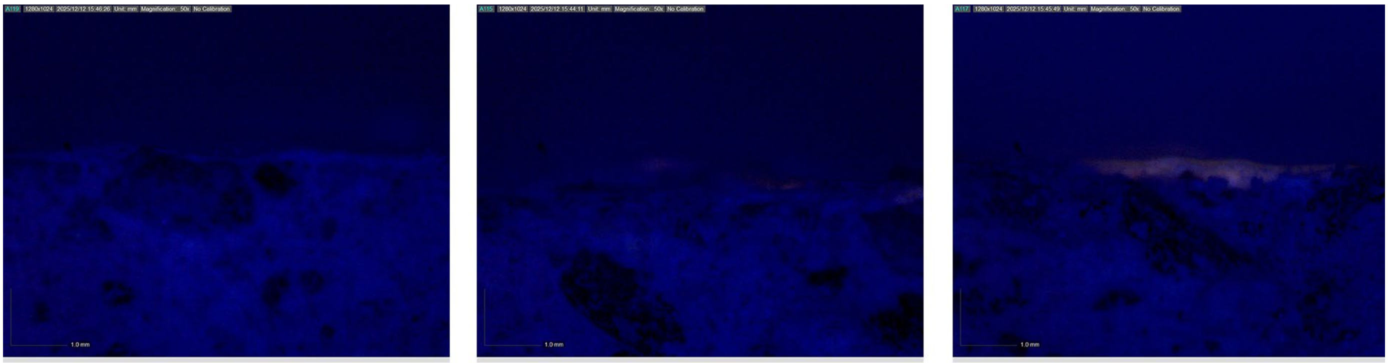

(d)

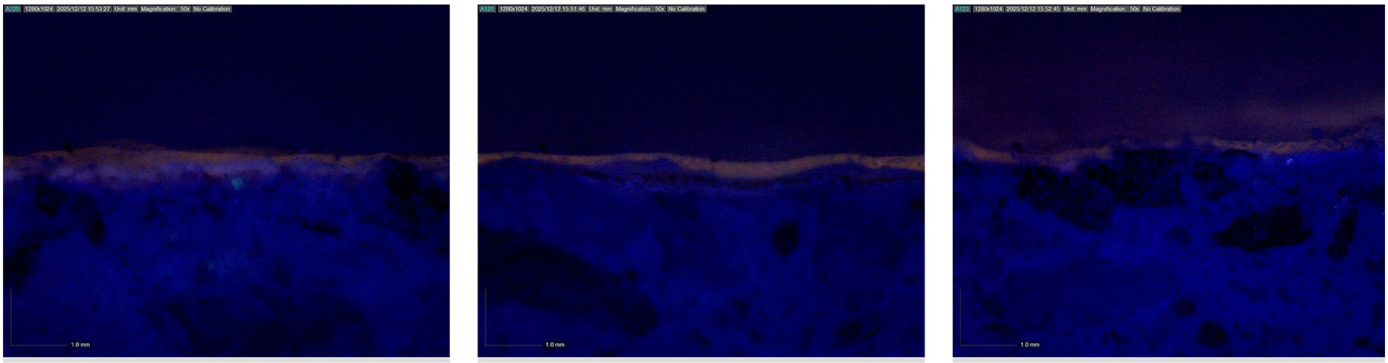

(e)

Figure S5. Cross-sections of fresco specimens at 50× magnification after cleaning. The acrylic layer, marked with a Rhodamine tracer, was removed using each delivery system in triplicate: (a) NS1, NS2, NS3; (b) NK1, NK2, NK3; (c) NV1, NV2, NV3; (d) AG1, AG2, AG3; (e) PB1, PB2, PB3.
